# High Influenza Incidence and Disease Severity Among Children and Adolescents Aged <18 Years ― United States, 2022–23 Season

**DOI:** 10.15585/mmwr.mm7241a2

**Published:** 2023-10-13

**Authors:** Elizabeth B. White, Alissa O’Halloran, Devi Sundaresan, Matthew Gilmer, Ryan Threlkel, Arielle Colón, Katie Tastad, Shua J. Chai, Nisha B. Alden, Kimberly Yousey-Hindes, Kyle P. Openo, Patricia A. Ryan, Sue Kim, Ruth Lynfield, Nancy Spina, Brenda L. Tesini, Marc Martinez, Zachary Schmidt, Melissa Sutton, H. Keipp Talbot, Mary Hill, Matthew Biggerstaff, Alicia Budd, Shikha Garg, Carrie Reed, A. Danielle Iuliano, Catherine H. Bozio

**Affiliations:** ^1^Influenza Division, National Center for Immunization and Respiratory Diseases, CDC; ^2^Epidemic Intelligence Service, CDC; ^3^Goldbelt Professional Services, Chesapeake, Virginia; ^4^California Emerging Infections Program, Oakland, California; ^5^Office of Readiness and Response, CDC; ^6^Colorado Department of Public Health & Environment; ^7^Connecticut Emerging Infections Program, Yale School of Public Health, New Haven, Connecticut; ^8^Georgia Emerging Infections Program, Georgia Department of Public Health, Atlanta, Georgia; ^9^Emory University School of Medicine, Atlanta, Georgia; ^10^Atlanta Veterans Affairs Medical Center, Decatur, Georgia;^ 11^Maryland Department of Health; ^12^Michigan Department of Health & Human Services; ^13^Minnesota Department of Health; ^14^New York State Department of Health; ^15^University of Rochester School of Medicine and Dentistry, Rochester, New York; ^16^New Mexico Department of Health; ^17^Ohio Department of Health; ^18^Public Health Division, Oregon Health Authority, Portland, Oregon; ^19^Vanderbilt University Medical Center, Nashville, Tennessee; ^20^Salt Lake County Health Department, Salt Lake City, Utah.

**Summary**

**What is already known about this topic?**

The 2022–23 influenza season began early, coinciding with circulation of other respiratory viruses. High hospitalization rates among children and adolescents were observed.

**What is added by this report?**

Among children and adolescents aged <18 years, 2022–23 was a high severity influenza season compared with thresholds based on previous seasons’ data; influenza-associated medical visits and hospitalizations met or exceeded incidence in previous seasons.

**What are the implications for public health practice?**

CDC recommends that all persons aged ≥6 months without contraindications should receive the annual seasonal influenza vaccine, ideally by the end of October.

## Abstract

During the 2022–23 influenza season, early increases in influenza activity, co-circulation of influenza with other respiratory viruses, and high influenza-associated hospitalization rates, particularly among children and adolescents, were observed. This report describes the 2022–23 influenza season among children and adolescents aged <18 years, including the seasonal severity assessment; estimates of U.S. influenza-associated medical visits, hospitalizations, and deaths; and characteristics of influenza-associated hospitalizations. The 2022–23 influenza season had high severity among children and adolescents compared with thresholds based on previous seasons’ influenza-associated outpatient visits, hospitalization rates, and deaths. Nationally, the incidences of influenza-associated outpatient visits and hospitalization for the 2022–23 season were similar for children aged <5 years and higher for children and adolescents aged 5–17 years compared with previous seasons. Peak influenza-associated outpatient and hospitalization activity occurred in late November and early December. Among children and adolescents hospitalized with influenza during the 2022–23 season in hospitals participating in the Influenza Hospitalization Surveillance Network, a lower proportion were vaccinated (18.3%) compared with previous seasons (35.8%–41.8%). Early influenza circulation, before many children and adolescents had been vaccinated, might have contributed to the high hospitalization rates during the 2022–23 season. Among symptomatic hospitalized patients, receipt of influenza antiviral treatment (64.9%) was lower than during pre–COVID-19 pandemic seasons (80.8%–87.1%). CDC recommends that all persons aged ≥6 months without contraindications should receive the annual influenza vaccine, ideally by the end of October.

## Introduction

During the 2022–23 season, influenza activity in the United States began in early October, earlier than in most previous seasons, and returned to pre–COVID-19 levels (*1*). In addition, high pediatric influenza hospitalization rates in the southeast (*2*), co-circulation of influenza virus with SARS-CoV-2 and respiratory syncytial virus (RSV), and a limited reduction in the availability of the influenza antiviral medication oseltamivir* were observed. Each year, CDC assesses seasonal severity by comparing current season’s influenza activity with thresholds based on peak influenza activity in previous seasons (*3*) and estimates the numbers and rates of influenza-associated medical visits, hospitalizations, and deaths in the United States (*4*). This report describes the 2022–23 influenza season among children and adolescents, including seasonal severity, estimated incidence, and characteristics of hospitalized patients. This analysis focuses on the 2022–23 influenza season compared with 2016–17 through 2021–22, excluding 2020–21 (during the peak of the COVID-19 pandemic) when influenza activity was minimal.

## Methods

CDC classifies each influenza season’s severity using three indicators. First, the percentage of all outpatient visits for influenza-like illness (ILI), defined as fever plus cough or sore throat, is obtained from the U.S. Outpatient Influenza-like Illness Surveillance Network (ILINet) (*5*). Second, rates of laboratory-confirmed influenza hospitalization^†^ are estimated through the Influenza Hospitalization Surveillance Network (FluSurv-NET)^§^^,^^¶^ (*6*). Finally, the percentage of all deaths due to influenza is calculated from National Vital Statistics System death registry data** (*5*). For each severity indicator, 50th, 90th, and 98th percentile intensity thresholds (ITs) are calculated from a distribution based on the geometric mean of peak weekly values in previous seasons^††^ (*3*,*7*). Seasonal severity is classified as low if at least two of the three indicators peak below IT_50_, and as moderate, high, or very high if at least two of the three indicators peak above IT_50_, IT_90_, or IT_98_, respectively.

The incidence of influenza-associated outpatient visits, hospitalizations, and deaths is estimated each season and is presented in this report as events per 100,000 population. Influenza-associated hospitalizations are estimated by applying the FluSurv-NET hospitalization rates, after adjustment for possible underdetection based on the probability of being tested and diagnostic test sensitivity,^¶^ to the U.S. population. To estimate influenza-associated outpatient visits, the ratio of outpatient illnesses to hospitalizations and the proportion of those with ILI who seek care are applied to the hospitalization estimates. To estimate influenza-associated deaths, the ratio of hospitalizations to deaths is applied to the hospitalization estimates (*4*,*8*). Pediatric rates are estimated for children aged <5 years and for children and adolescents aged 5–17 years.

Characteristics of influenza-associated hospitalizations, including influenza vaccination status,^§§^ were abstracted from medical charts by trained FluSurv-NET surveillance staff members using a standard case report form. Data were collected for all hospitalized children and adolescents across the 2016–17 through 2021–22 seasons and for an age-stratified sample during the 2022–23 season. Weighted proportions are reported overall and by age group (<5 and 5–17 years).

This analysis presents preliminary 2022–23 season data,^¶¶^ reported as of September 21, 2023, among children and adolescents, compared with data from 2016–17 through 2021–22 (excluding 2020–21). Analyses were conducted using R (version 4.1.2; R Foundation) and SAS (version 9.4; SAS Institute). This activity was reviewed by CDC, deemed not research, and was conducted consistent with applicable federal law and CDC policy.*** FluSurv-NET sites obtained human subjects and ethics approval from their respective state health department, academic partner, and participating hospital institutional review boards.

## Results

**FIGURE 1:**
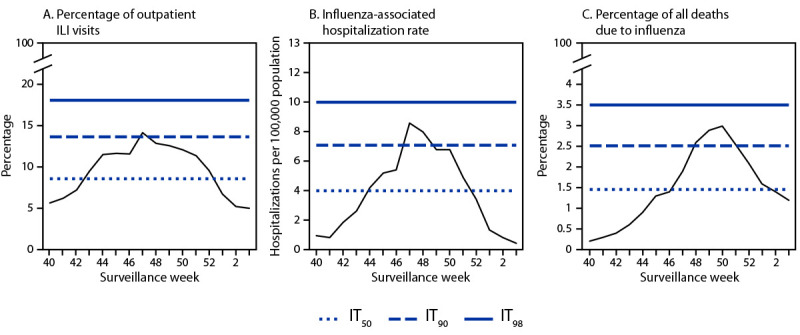
Percentage of outpatient visits for influenza-like illness (A), influenza-associated hospitalization rates (B), and percentage of all-cause deaths due to influenza (C) among children and adolescents aged <18 years — United States, 2022–23 influenza season* **Abbreviations:** FluSurv-NET = Influenza Hospitalization Surveillance Network; ILI = influenza-like illness; ILINet = Outpatient Influenza-like Illness Surveillance Network; IT = intensity threshold; NCHS = National Center for Health Statistics. * In 2022–23, ITs for the percentage of outpatient visits for ILI were calculated using ILINet data from 2016–17 through 2021–22, excluding 2020–21. ITs for influenza-associated hospitalizations were calculated using FluSurv-NET data from 2006–07 through 2021–22, excluding 2011–12 and 2020–21. ITs for the percentage of deaths due to influenza were calculated using NCHS data from 2010–11 through 2021–22, excluding 2011–12 and 2020–21.

For children and adolescents, the 2022–23 influenza season was classified as high severity, with the weekly percentage of outpatient visits for ILI, influenza-associated hospitalization rate, and percentage of deaths due to influenza all peaking between IT_90_ and IT_98_ (Figure 1). The percentage of outpatient visits that were for ILI and the rate of influenza-associated hospitalizations peaked in late November 2022, 3 weeks before the percentage of influenza deaths peaked; deaths remained high for 4 weeks in December 2022.

**FIGURE 2:**
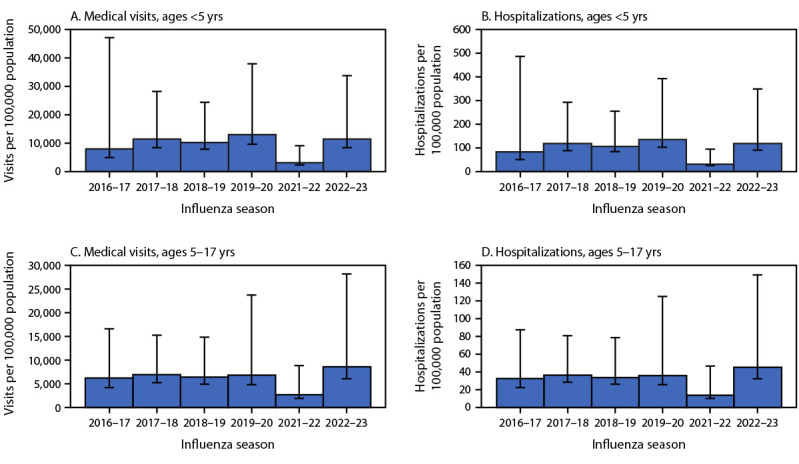
Influenza-associated medical visits* (A and C) and influenza-associated hospitalizations* (B and D) among children aged <5 years and children and adolescents aged 5–17 years — United States, 2016–17 through 2022–23 influenza seasons^†^ * With 95% credible intervals indicated by error bars. ^†^ Excluding 2020–21.

Nationally, point estimates of the rates of influenza-associated medical visits, hospitalization, and death estimated during the 2022–23 season were higher among children aged <5 years than among children and adolescents aged 5–17 years (Figure 2). However, among children and adolescents aged 5–17 years, rates of influenza-associated medical visits and hospitalizations were higher during 2022–23 than any season since 2016–17. Children aged <5 years had the second highest rates of influenza-associated medical visits (11,443 per 100,000) and hospitalization (119 per 100,000) in 2022–23 since the 2016–17 season. Rates of influenza-associated deaths in 2022–23 were low and consistent with previous seasons: 1.2 per 100,000 among children <5 and 0.5 per 100,000 among children and adolescents aged 5–17 years (Supplementary Figure, https://stacks.cdc.gov/view/cdc/133678).

During October 1, 2022–April 30, 2023, FluSurv-NET identified 2,762 influenza-associated hospitalizations in children and adolescents aged <18 years, 2,108 of which were sampled and had clinical data available. The median age was 5 years (IQR = 2–9 years), 57.4% were male, and 50.5% had an underlying condition, similar to recent seasons (Table). The most common underlying medical conditions were asthma, neurologic disorders, and obesity. Most (95.4%) infections were with influenza A virus; 80.2% of those subtyped were A(H3N2) and 19.6% were A(H1N1)pdm09. More than one half (57.1%) of the 2022–23 season’s total pediatric hospitalizations occurred during October and November 2022, higher than the percentages occurring in October and November in the 2016–17 through 2021–22 seasons (1.6%–6.8%). Among hospitalized children and adolescents in 2022–23, 18.3% had received an influenza vaccine, compared with 35.8%–41.8% in 2016–17 through 2021–22. The proportion of pediatric patients with respiratory symptoms who received influenza antiviral treatment during their hospitalization in 2022–23 (64.9%) was similar to the proportion in 2021–22 (61.5%), but lower than that during pre–COVID-19 pandemic seasons (80.8%–87.1%). Among all pediatric hospitalizations, the proportions who were admitted to an intensive care unit (18.4%), who required invasive mechanical ventilation (4.7%), or who died in hospital (0.4%) were similar to the proportions during previous influenza seasons.

**TABLE:** Characteristics, treatment, and outcomes among children and adolescents hospitalized with laboratory-confirmed influenza, compared with 2016–17 through 2021–22,* overall and by age group — FluSurv-NET, 2022–23 influenza season

Characteristic	Age group, yrs					
	<18		<5		5–17	
	Weighted %, 2022–23	Range of unweighted %, 2016–17 through 2021–22	Weighted %, 2022–23	Range of unweighted %, 2016–17 through 2021–22	Weighted %, 2022–23	Range of unweighted %, 2016–17 through 2021–22
**Sex**						
Female	42.6	42.7–45.1	43.8	41.4–45.2	41.5	41.8–47.3
Male	57.4	54.9–57.3	56.2	54.9–58.6	58.5	52.7–58.2
**Race and ethnicity**						
American Indian or Alaska Native, non-Hispanic	1.3	1.0–1.6	1.4	0.8–1.9	1.1	0.3–1.3
Asian or Pacific Islander, non-Hispanic	5.9	4.1–6.4	6.9	4.5–7.0	5.0	2.7–5.7
Black or African American, non-Hispanic	29.0	24.6–35.0	26.3	24.4–33.2	31.7	24.9–36.8
White, non-Hispanic	31.8	31.1–34.3	28.9	28.4–31.3	34.7	33.9–39.4
Hispanic or Latino	23.8	22.2–27.0	27.1	18.8–29.5	20.4	15.4–23.7
Multiple races, non-Hispanic	1.8	1.2–1.6	1.5	0.4–1.7	2.0	0.7–1.9
**Influenza A**	95.4	48.9–98.0	95.5	52.4–94.2	94.5	43.9–98.6
**Influenza A subtype** ^†^						
A(H1N1)pdm09	19.6	0.5–95.5	18.5	1.1–96.7	20.8	0–93.4
A(H3N2)	80.2	4.5–99.5	81.4	3.3–98.9	79.1	6.6–100.0
**Received seasonal influenza vaccine** ^§^						
Yes	18.3	35.8–41.8	18.1	38.2–45.8	18.4	32.1–40.6
No	67.1	43.7–57.8	67.0	38.7–56.1	67.3	47.0–59.8
Unknown	14.6	5.8–20.5	14.9	5.5–19.8	14.3	6.2–21.0
**Underlying medical conditions**						
Any underlying medical condition^¶^	50.5	47.5–58.1	33.9	34.1–39.9	67.1	66.4–76.2
Asthma	27.6	22.9–29.2	14.9	13.5–16.3	39.7	33.6–43.4
Chronic lung disease	4.8	6.4–8.2	2.6	4.5–6.8	6.9	6.6–9.5
Chronic metabolic disease	5.0	4.1–6.2	1.8	2.0–4.5	8.0	6.0–9.5
Diabetes	2.4	1.1–3.5	0.4	0.2–0.8	4.3	2.3–5.7
Cardiovascular disease	6.8	5.0–8.8	6.8	6.1–8.6	6.8	4.0–9.6
Blood disorder	6.5	5.2–10.3	3.3	2.7–7.5	9.6	6.7–13.0
Immunocompromising condition	7.7	6.3–9.9	4.6	3.1–5.6	10.5	8.7–14.9
Liver disease	0.5	0.6–1.5	0	0.4–1.0	1.0	0.9–2.1
Neurologic disorder	15.7	15.6–20.1	13.0	11.4–15.7	18.2	21.4–24.4
Obesity	14.4	13.3–15.5	10.3	8.9–11.3	16.4	15.1–18.2
Renal disease	1.5	1.7–2.3	0.7	0.4–1.6	2.4	2.2–3.9
**Treatment and outcomes**						
Received influenza antiviral treatment during hospitalization**	64.9	61.5–87.1	64.2	62.3–87.8	65.7	60.7–86.0
Admitted to intensive care unit	18.4	21.2–22.8	17.5	20.2–22.4	19.4	21.9–24.3
Invasive mechanical ventilation	4.7	4.7–5.7	3.7	4.0–5.3	5.6	4.5–6.3
Died in hospital	0.4	0–0.6	0.4	0–0.6	0.5	0–1.1

* Excluding 2020–21.

^†^ Among 49% of Influenza A specimens that were subtyped.

^§^ Excluding children aged <6 months, who are not eligible to receive influenza vaccine.

^¶^ At least one of the following conditions: asthma, chronic lung disease, chronic metabolic disease (e.g., diabetes), blood disorder, cardiovascular disease, neurologic disorder, immunocompromising condition, obesity, renal disease, or liver disease.

** Restricted to patients with respiratory symptoms.

## Discussion

The 2022–23 influenza season was classified as high severity among children and adolescents, the fourth season with that classification since the 2009 influenza A(H1N1) pandemic. Further, all three severity indicators not only surpassed intensity levels for high severity, but the peaks also occurred early in the season (late November and early December) (*1*). National estimates of the rates of influenza-associated medical visits and hospitalizations were higher than those during most previous seasons for children aged <5 years and children and adolescents aged 5–17 years. This high incidence strained health care systems, particularly with the co-circulation of SARS-CoV-2 and RSV.^†††^

Among children and adolescents hospitalized with influenza during 2022–23, a substantially lower proportion were vaccinated compared with previous seasons, which could be related to low vaccination coverage in the population, high vaccine effectiveness, or both. The National Immunization Survey^§§§^ estimates that when pediatric influenza-associated hospitalization rates peaked during the week ending November 26, 2022, only 41.9% of children and adolescents aged 6 months–17 years nationwide had received their annual influenza vaccination (compared with 55.1% by the end of the season). Influenza vaccination coverage by the end of November was similar in 2022 and in 2021 (45.0%), but lower than in 2019 (51.9%) and 2020 (49.7%). Preliminary assessments have shown that the 2022–23 influenza vaccine provided moderately strong (68%) protection against pediatric hospitalization.^¶¶¶^ The combination of low influenza vaccine coverage early in the season and unusually early influenza activity (57.1% of the season’s pediatric hospitalizations occurred by the end of November) likely contributed to the high observed rate of influenza-associated hospitalization, despite the moderately strong protection from the 2022–23 influenza vaccine. In addition, a lower proportion of symptomatic hospitalized patients in 2022–23 received influenza antiviral medication compared with that during pre–COVID-19 pandemic seasons. Taken together, these findings underscore the importance of children and adolescents receiving a seasonal influenza vaccination, ideally by the end of October (*9*), and prompt influenza antiviral treatment for those who are hospitalized.****

### Limitations

The findings in this report are subject to at least five limitations. First, within FluSurv-NET, influenza testing was performed at the clinician’s discretion or based on facility-level practices, which might affect the observed clinical epidemiology of influenza-associated hospitalizations. Second, severity assessment and incidence estimation adjustments for the frequency of influenza testing and other ratios were based on previous seasons’ data and might not reflect current testing practices or health care–seeking behaviors. Third, FluSurv-NET catchment areas cover approximately 9.0% of the U.S. population; characteristics of children and adolescents hospitalized with influenza might not be generalizable to all pediatric hospitalizations in the United States. Fourth, historical data used for the severity assessment might be a suboptimal comparison if recent influenza activity differs from that before the COVID-19 pandemic; classifications of being above or between threshold levels are qualitative and do not reflect statistical differences. Finally, comparisons of rates of influenza-associated outpatients visits, hospitalizations, and deaths across seasons based on point estimates are descriptive and intended to highlight trends, not statistical differences. 

### Implications for Public Health Practice

The 2022–23 influenza season was classified as high severity for children and adolescents based on influenza-associated outpatient visits, hospitalization rates, and deaths. Among hospitalized children and adolescents with influenza, receipt of influenza vaccine was lower than that during previous seasons, which might have been in part related to most influenza hospitalizations occurring earlier. The proportion of pediatric hospitalizations treated with influenza antiviral medication was lower than in pre–COVID-19 pandemic seasons; prompt antiviral treatment is important for symptomatic patients hospitalized with influenza. All persons aged ≥6 months are recommended by CDC to receive the annual seasonal influenza vaccine, ideally by the end of October.

## Footnotes

*
https://emergency.cdc.gov/han/2022/han00482.asp

† A FluSurv-NET patient was defined as a person who 1) was a resident of the surveillance catchment area, 2) had a hospital admission during October 1–April 30 of a given season, and 3) received a positive influenza test result ≤14 days before or anytime during hospitalization.

§ FluSurv-NET is a population-based surveillance network for influenza-associated hospitalizations. The 2022–23 season included data from selected counties in 13 U.S. states, covering approximately 9% of the U.S. population: California, Colorado, Connecticut, Georgia, Maryland, Michigan, Minnesota, New Mexico, New York, Ohio, Oregon, Tennessee, and Utah.

¶ Within hospitals participating in FluSurv-NET, influenza testing is performed at the clinician’s discretion or based on facility-level practices. For the severity assessment and incidence estimates, influenza-associated hospitalization rates were adjusted for influenza testing practices and diagnostic test sensitivity to account for possible underdetection of influenza test–positive hospitalizations.

** The National Center for Health Statistics (NCHS) collects death certificate data from state vital statistics offices for all deaths occurring in the United States. Deaths included in the U.S. Influenza Surveillance System are those classified based on *International Classification of Diseases, Tenth Revision* (ICD-10) cause of death codes as associated with influenza, COVID-19, or pneumonia. Data are aggregated by the week of death occurrence. To avoid bias arising from COVID-19, counts of NCHS ICD-10–coded influenza deaths were used instead of pneumonia and influenza deaths to calculate mortality thresholds and assess severity.

†† ITs for the percentage of outpatient visits for ILI were calculated using ILINet data from 2016–17 through 2021–22, excluding 2020–21. ITs for influenza-associated hospitalizations were calculated using FluSurv-NET data from 2006–07 through 2021–22, excluding 2011–12 and 2020–21. ITs for the percentage of deaths due to influenza were calculated using NCHS data from 2010–11 through 2021–22, excluding 2011–12 and 2020–21.

§§ A patient was considered to have received the current seasonal influenza vaccine if ≥1 dose was administered ≥14 days before receipt of a positive influenza test result. Ascertainment of vaccination status was performed using hospital records, state immunization registries, primary care provider surveys, and patient or proxy interview.

¶¶ FluSurv-NET data, as well as incidence and severity estimates, are current as of September 21, 2023. Incidence and severity analyses are finalized once data on testing practices are available, usually 2 years after the end of the season. Incidence estimates and severity assessment interpretations might change.

*** 45 C.F.R. part 46.102(l)(2), 21 C.F.R. part 56; 42 U.S.C. Sect. 241(d); 5 U.S.C. Sect. 552a; 44 U.S.C. Sect. 3501 et seq.

†††
https://emergency.cdc.gov/han/2022/pdf/CDC_HAN_479.pdf

§§§ https://www.cdc.gov/flu/fluvaxview/dashboard/vaccination-dashboard.html (Accessed September 21, 2023).

¶¶¶
https://www.cdc.gov/flu/vaccines-work/2022-2023.html

****
https://www.cdc.gov/flu/professionals/antivirals/summary-clinicians.htm#summary
